# An efficient and robust MRI-guided radiotherapy planning approach for targeting abdominal organs and tumours in the mouse

**DOI:** 10.1371/journal.pone.0176693

**Published:** 2017-04-28

**Authors:** Veerle Kersemans, John S. Beech, Stuart Gilchrist, Paul Kinchesh, Philip D. Allen, James Thompson, Ana L. Gomes, Zenobia D’Costa, Luke Bird, Iain D. C. Tullis, Robert G. Newman, Aurelien Corroyer-Dulmont, Nadia Falzone, Abul Azad, Katherine A. Vallis, Owen J. Sansom, Ruth J. Muschel, Borivoj Vojnovic, Mark A. Hill, Emmanouil Fokas, Sean C. Smart

**Affiliations:** 1 Cancer Research UK and Medical Research Council Oxford Institute for Radiation Oncology, Department of Oncology, University of Oxford, Oxford, United Kingdom; 2 Cancer Research UK Beatson Institute, Glasgow, United Kingdom; 3 Department of Radiotherapy and Oncology, Goethe University Frankfurt, Frankfurt, German; 4 German Cancer Research Center (DKFZ), Heidelberg, Germany, German Cancer Consortium (DKTK) (Partner Site), Frankfurt, Germany; North Shore Long Island Jewish Health System, UNITED STATES

## Abstract

**Introduction:**

Preclinical CT-guided radiotherapy platforms are increasingly used but the CT images are characterized by poor soft tissue contrast. The aim of this study was to develop a robust and accurate method of MRI-guided radiotherapy (MR-IGRT) delivery to abdominal targets in the mouse.

**Methods:**

A multimodality cradle was developed for providing subject immobilisation and its performance was evaluated. Whilst CT was still used for dose calculations, target identification was based on MRI. Each step of the radiotherapy planning procedure was validated initially in vitro using BANG gel dosimeters. Subsequently, MR-IGRT of normal adrenal glands with a size-matched collimated beam was performed. Additionally, the SK-N-SH neuroblastoma xenograft model and the transgenic KPC model of pancreatic ductal adenocarcinoma were used to demonstrate the applicability of our methods for the accurate delivery of radiation to CT-invisible abdominal tumours.

**Results:**

The BANG gel phantoms demonstrated a targeting efficiency error of 0.56 ± 0.18 mm. The in vivo stability tests of body motion during MR-IGRT and the associated cradle transfer showed that the residual body movements are within this MR-IGRT targeting error. Accurate MR-IGRT of the normal adrenal glands with a size-matched collimated beam was confirmed by γH2AX staining. Regression in tumour volume was observed almost immediately post MR-IGRT in the neuroblastoma model, further demonstrating accuracy of x-ray delivery. Finally, MR-IGRT in the KPC model facilitated precise contouring and comparison of different treatment plans and radiotherapy dose distributions not only to the intra-abdominal tumour but also to the organs at risk.

**Conclusion:**

This is, to our knowledge, the first study to demonstrate preclinical MR-IGRT in intra-abdominal organs. The proposed MR-IGRT method presents a state-of-the-art solution to enabling robust, accurate and efficient targeting of extracranial organs in the mouse and can operate with a sufficiently high throughput to allow fractionated treatments to be given.

## Introduction

It is prerequisite, when translating preclinical findings to the clinic, that the animal models and techniques should mimic aspects of the human disease and treatment faithfully [1]. For simple superficial tumours, including xenografts, radiotherapy (RT) dose prescription and delivery is typically based upon wide-field irradiation with visually-guided lead shielding of tissues to remain unirradiated [2]. Where more complicated, but increasingly clinically relevant, orthotopic and genetically engineered mouse models are used this is not an option as the tumours are often internal, and visual guidance is not possible [3–5]. This limitation is being addressed by preclinical image-guided radiation treatment (IGRT) platforms, recently developed to mimic the clinical paradigm of irradiation planning and delivery [6].

An overview of the small animal IGRT research platforms was presented by Verhaegen et al in which it is stated that the majority of radiotherapy platforms are paired with x-ray computed tomography (CT)[6]. This replicates the clinical situation and enables dose estimation via CT imaging of the electron density of each tissue type. Unfortunately, in the case of low energy small animal irradiators, CT provides an inherently poor contrast between soft tissues [7]. This poses a significant problem for abdominal organs, orthotopic tumours and even some xenografts since meaningful delivery of radiotherapy is difficult without correct localisation and delineation of the target. Furthermore, the estimation of dose distribution to the organs at risk (OARs), especially for intra-abdominal tumours is practically impossible.

To attempt to overcome the disadvantages of CT, another strategy, termed reverse-contrast imaging, has been proposed for enabling RT of pancreatic cancer [8]. Here, CT was used for IGRT but with the intraperitoneal space filled with CT contrast agent. Although physical separation of the abdominal structures was achieved, the accuracy of dose estimation remained limited [6]. Other imaging modalities such as Positron Emission Tomography (PET) and Bioluminescence imaging (BLI) also have been proposed to guide targeting and delivery of radiotherapy [2, 9–11] but both techniques have their inherent limitations. BLI is restricted by its light penetration depth, lack of true tomography imaging and requires the use of reporter genes whilst PET applications are also limited by partial volume effects and in some cases, inadequate contrast specificity. In contrast to CT, Magnetic Resonance Imaging (MRI) provides exquisite soft tissue contrast that can often be optimised for the organ of interest. However, MRI has to date been used merely as a descriptor of target location [2, 12] in studies involving organs other than the brain. MRI-guided radiotherapy (RT) has only focussed on brain, an organ that is easy to immobilise with internal, fixed landmarks [2, 9, 11, 12]. As such, the skull’s bright intensity on CT but dark intensity on MRI was used to drive a rigid body registration. This approach is aided by the fact that the skull encapsulates that target tissue of interest. Moreover, the brain will remain relatively stationary within the skull even where gross body deformations occur in-between MR and CT acquisitions. In contrast, within the body such a rigid, encapsulating boundary to drive the registration does not exist and other solutions must be sought. More recently, CT was omitted in a MRI-only RT workflow but, by doing so, its usefulness was restricted to the rat brain as only limited tissue classes were available for accurate dose planning. Furthermore a common coordinate system between the MR and RT systems was missing [11].In short no general solution has been proposed to accurately target defined anatomical structures that are not resolved upon CT imaging.

The aim of this study was to develop a robust and accurate method of MRI-guided radiotherapy (MR-IGRT) delivery to abdominal targets in the mouse. To achieve this, subject immobilisation throughout the whole procedure is crucial as accurate registration of MRI-to-CT images will depend on the magnitude of intra- and/or inter-scan displacements [10, 13]. A multimodality cradle was therefore developed, enabling transfer of the mouse without the introduction of large scale intra-subject motion between any of our imaging systems (MRI at 4.7T, 7.0T and 9.4T, PET, CT and Single Photon Emission Computed Tomography) and the IGRT platform. Whilst central to the deployment of the proposed MR-IGRT technique, the animal support cradle, which incorporated anaesthetic gas delivery, rectal temperature maintenance and respiratory monitoring, solved only part of the requirement. In addition, newly-developed rapid and respiratory motion desensitised T2-weighted MRI imaging techniques were used together with a deformable registration technique, based on the one described by Heinrich et al., [14] to guide the MR-CT registration and to localise the tumours or organs of interest.

Each step of the radiotherapy planning process was validated, both in vitro using BANG gel dosimeters and in vivo using different animal models. The in vitro tests were based on clinical routine where the systematic accuracy of RT delivery is tested by using radiosensitive polymer gels to validate 3-dimensional dosimetry for brachytherapy treatment planning, conformal and intensity modulated radiotherapy techniques and stereotactic radiosurgery [13–15]. These gels polymerise following exposure to ionising radiation, and the resulting absorbed radiation dose fixated in the polymer gel can be read out by several imaging techniques, including MRI. As such they can be used to measure the 3D dose distributions of geometrically complex radiotherapy dose schemes. An overview of their history and working mechanism is presented elsewhere [15]. Such gels are commercially available and can be delivered ready-for-use, thus avoiding the intricate manufacturing process and giving highest MR sensitivity upon irradiation [16, 17]. This method provides a tool for simulating the entire treatment procedure and offers high-resolution 3D dose distribution measurements.

Next, in vivo models with increasing complexity and clinical relevance were used to validate the MR-IGRT workflow in vivo. MR-IGRT of single adrenal glands and subsequent histological staining based on γH2AX imaging was performed to confirm selective targeting in vivo. The SK-N-SH neuroblastoma xenograft model was used to demonstrate the applicability of our methods for evaluation of response-to-treatment. Finally, the applicability of MRI-guided RT planning for use in abdominal tumours was demonstrated using the KPC model of pancreatic ductal adenocarcinoma (PDAC)[15].

## Materials and methods

The overall workflow and design of the study is presented as a flowchart in the supplementary data S1 Fig.

### Animals

#### Ethics statement

All procedures were conducted in accordance with the Animals Scientific Procedures Act of 1986 (UK) (Project License Numbers 30/3059, 30/2922 and 30/3266 issued by the Home Office). The protocol was approved by the Committee on the Ethics of Animal Experiments of the University of Oxford.

#### Animals and husbandry

CBA/CaCrl mice (*Mus Musculus*) (Charles River, UK) were used for assessments of body motion and for the in vivo testing of MR-IGRT to the adrenal glands. BalbC nude mice (Charles River, UK) were used for examination of the response to MR-IGRT in a xenograft model of neuroblastoma. KPC (K-ras^LSL.G12D/+;^ p53^R172H/+;^ Pdx^Cretg/+)^ mice (Beatson Institute, Glasgow, UK) were bred in the biomedical services unit at the University of Oxford and were used to examine the potential of MR-IGRT in a spontaneous model of pancreatic cancer [16].

All mice were housed in individual ventilated cages in a separate room with 12-h dark and light cycle maintained at 22°C in 50% humidity. They were provided with certified rodent diet, filtered water ad libitum, autoclaved bedding material and cage enrichment. No mice had to be euthanized on welfare grounds.

#### Anaesthesia for imaging and RT

Anaesthesia was induced and maintained using isoflurane (1–4%) in room air supplemented with oxygen (80%/20% v/v). Animals were placed in head-first supine position for imaging. Animals were either recovered afterwards with no ill effect, or culled humanely in the case of in vivo targeting validation. Throughout imaging experiments, mice were maintained at 37°C core temperature, respiration rate was monitored and maintained in the range of 40–60 breaths/minute.

### Animal support apparatus

#### Multi-modal cradle design

**Fig 1:**
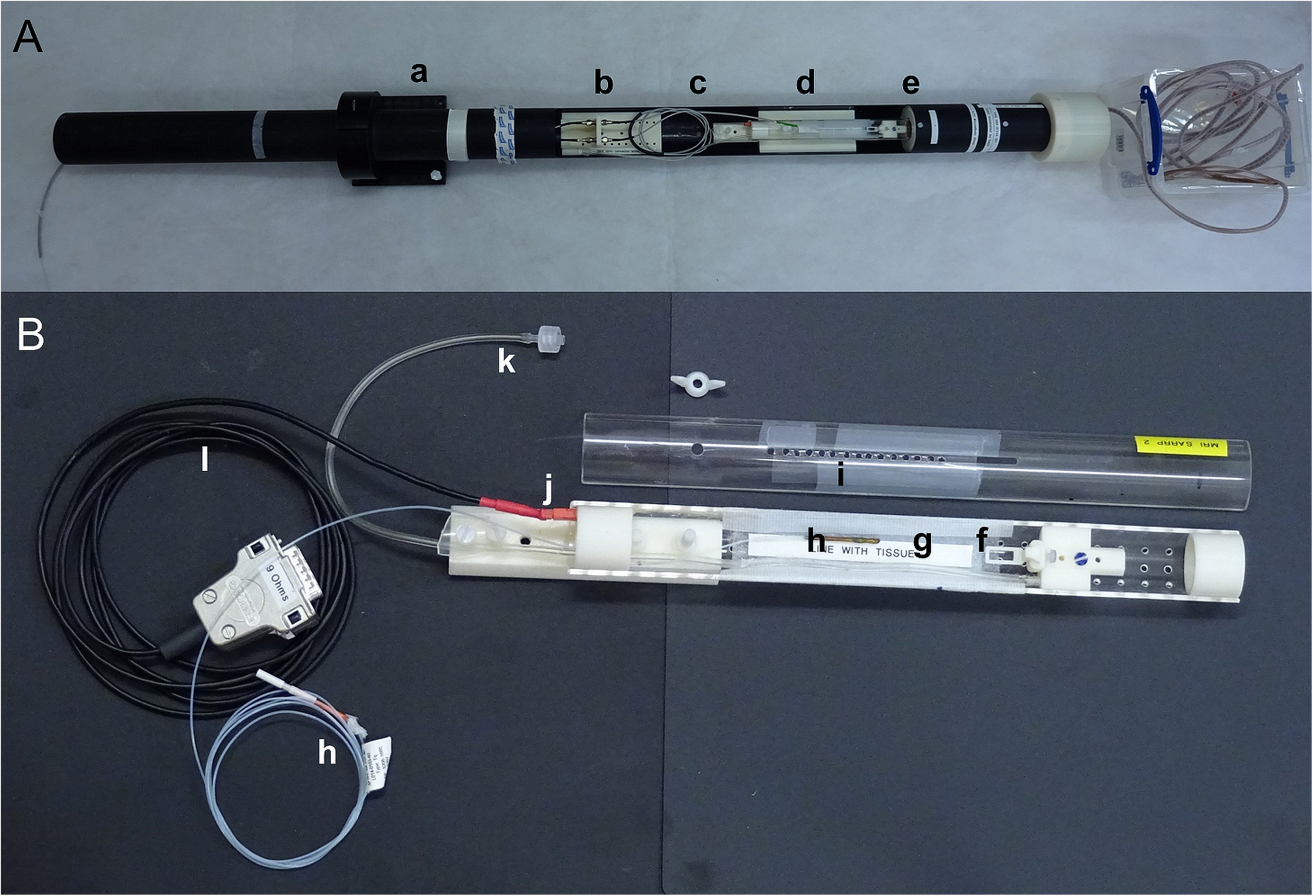
Photograph of the animal support cradle. (A) Animal support cradle mounted in the MRI cradle assembly showing (a) MRI support cradle, (b) mounting block for the optical fibres for respiration monitoring, (c) optical fibres for respiration monitoring, (d) trapezoidal base of the animal support cradle which mated with a support block on the MRI support cradle, (e) RF coil. (B) Close-up of animal support cradle showing (f) mouthpiece assembly consisting of a base block, a vertical and horizontal adjustable mouth bar and an anaesthetic gas delivery tube, (g) electrical heating blanket, (h) optical fibre for temperature monitoring, (i) top of the animal support cradle with access points for the optical fibres for respiration monitoring, (j) quick-fit connector for electrical heating pad, (k) anaesthetic gas delivery tube with quick-fit connector and (l) power cable for the electrical heat delivery.

A custom-made MR and CT compatible animal support cradle was assembled as shown in Fig 1. Two 300 mm long acrylic tubes (i.d. 25 mm, o.d. 30 mm; The Plastic Shop, UK) were cut along the axis to give two halves that formed a cylinder when pushed together. The mouthpiece assembly consisted of a base block, a vertically and horizontally adjustable mouth bar and an anaesthetic gas delivery tube. The bottom half-tube was screwed to a trapezoidal base which mated with a support block used as part of the MRI support cradle. The top half-tube was supported on a ring and a locator screw mounted on the bottom half-tube. For cone beam CT (CBCT) imaging and subsequent radiotherapy using the IGRT system, the bottom half-tube was mated with a support block mounted on top of the robotic stages such that the cradle was kept within the 190 mm radius to prevent collisions resulting from rotation of the cradle while obtaining the CBCT image. Individual components were drawn using CAD software (Solidworks, Dassault Systèmes) and printed using a 3D printer (HP Designjet 3D).

#### Temperature maintenance

Core body temperature was monitored using a rectal OTP-M high accuracy temperature sensor (part number OTP-M-X-62F2.5–1.5(PTFE/PVC)-XN-7GT-M1-PV0014a, Opsens Industrial) connected via a 10 m fibre-optic extension to an AccuSens 4 channel Signal Conditioner (part number ACS-P4-N-62SC). A thin layer of epoxy adhesive was applied over the last few millimetres of the probe to smooth over any sharp edges that could injure the animal. An analogue output voltage available from the signal conditioner was used to control the heating circuit. Temperature was maintained at 37°C core temperature using a custom-made homeothermic electrical driver unit based around a programmable controller unit (Arduino) which delivered current into an MR-compatible twisted pair wire resistor [17]. The heating blanket was positioned beneath the animal and below the plane of rotation of the CT imaging gantry to avoid overt metal streak artefacts in the reconstructed CT images.

#### Respiration monitoring and generation of respiratory control signals for gated imaging

Respiration was monitored using a custom-made fibre optic reflection displacement sensor which uses closely spaced transmission and reception polymer optical fibres. These were placed through apertures spaced along the length of the top half-tube such that they faced a moving part of the body, due to respiration and in the expected vicinity of the target tissue to be irradiated.

The analogue optical displacement signal indicating respiratory motion was passed through a commercial gating control unit (DTU 200, Biopac Inc. CA). This unit forms a threshold-defined binary logic signal that instructs the MR scanner, in real time, whether to acquire data or not. Since some change in the signal level is required in order for the threshold to be reached (along with hysteresis to avoid repetitive noise triggering), some respiratory-derived data corruption is to be expected unless additional measures are taken. For the trailing exhalation edge, the respiration motion artefact was minimized by passing the thresholded (logic) signal through a monostable and gating circuit that extended, by a user variable delay time, the duration of the logic signal used to drive the scanner without data acquisition. In order to avoid respiration motion artefact from the leading, inhalation, edge of the breath, the MR scanner was programmed to reacquire a user-variable number of data blocks immediately after the same breath.

#### Transfer between MRI and IGRT system

The heater power cable, the optical fibres for respiration and temperature monitoring, and the tubing for anaesthetic gas delivery had ‘quick-fit’ connectors located at the animal cradle. The animal in its cradle could be thus quickly and easily detached from the physiological support apparatus located at the MRI scanner, transported to the IGRT system and re-connected to the IGRT physiological support system. Both the MRI scanner and IGRT system had their own independent, duplicated physiological monitoring and maintenance systems which were based around a commercial physiological monitoring system (MP150, Biopac Inc., CA).

### Mechanical accuracy testing using BANG polymer gel dosimeters

The in vitro target testing was performed to demonstrate that there were no systematic errors consequent to (a) the combination of images acquired using fundamentally different localisation techniques and (b) the imaging systems, produced by 2 different manufacturers, with no previous reference to each other.

#### Preparation of Rotationally-asymmetric BANG gels

Under anoxic conditions in a hypoxia chamber (INVIVO2 300, Ruskinn, Bridgend, UK), BANG gels (MGS Research Inc, Madison, USA) were melted at 37˚C and placed in glass vials that were angled such that, when the gels set, the meniscus was not horizontal [18]. The BANG gels were allowed to equilibrate to room temperature before imaging and radiotherapy was initiated. The BANG gels were stored in the dark at 4°C.

#### RT planning CBCT image

CT was performed using the standard cone beam CT (CBCT) imaging component of the Small Animal Radiation Research Platform (SARRP, Xstrahl, Surrey, UK). CBCT images were acquired using 70 kVp (BANG gels) X-rays (fine focus, 0.15 mm Cu filter) with 720 projections taken as the sample is rotated through 360° in a scan time of 127 s. Additional filtration results from the presence of a transmission monitor (in total: 0.105 mm Cu and 1 mm of FR4 a fibreglass/epoxy mix). This CT acquisition will be referred to as ‘RT planning CBCT image’. CBCT images were reconstructed using a modified Feldkamp-Davis-Kress reconstruction algorithm into 0.16 mm isotropic voxels. The dose per CBCT scan was typically 56.9 ± 2.8 mGy 70 kVp CT imaging, and was determined using EBT3 gafchromic film (Vertec Scientific Ltd) sandwiched in the middle of a 25.4 mm diameter water-equivalent (WT1; Barts and the London NHS Trust) cylinder as described before [19].

#### RT targeting

Therapeutic exposures were performed using 220 kVp X-rays (broad focus, 0.15 mm Cu filter and a half value layer of 0.93 mm Cu) delivered using the SARRP irradiator in conjunction with collimators to produce a range of field sizes. Additional filtration results from the presence of a transmission monitor (in total: 0.105 mm Cu and 1 mm of FR4 a fibreglass/epoxy mix). For each collimator, dosimetry was performed using an EBT3 film as described before, with the film calibrated following the recommendations of the report of the American Association of Physicists in Medicine Task Group 61 [20, 21]. Treatment planning and subsequent beam delivery was performed using Muriplan (Xstrahl Ltd, Camberley, Surrey, UK), with targeting and subsequent segmentation performed using the CBCT image [22].

#### Delivery of intersecting RT beams to form a target for MR-IGRT

The rotationally-asymmetric gels (n = 5) were placed vertically in the IGRT system. Following the low dose RT planning CBCT image, the gels were irradiated with a pair of orthogonal 2 mm or 4 mm in diameter circular beams each with approximately 2 Gy. Approximately 4 Gy was thus delivered to the intersection of the beams which had a volume of 4.2 or 33.5 mm^3^. The intersection was positioned in a way such as to avoid mirror image symmetry.

#### Definition of a RT planning MR and CT image

MRI was performed at 4.7 T (VNMRS, Varian Inc., Palo Alto, CA) using a 40 mm ID quadrature birdcage coil (Rapid Biomedical GmbH, Rimpar, Germany). The BANG gels were scanned with a 3D CE-FAST sequence [23] with TR 3.672 ms, TE 1.836 ms, bandwidth 100 kHz, FOV 70×35×35 mm3, matrix 256×128×128 and a 40° degree, 64 μs hard pulse. This CE-FAST scan was used as it showed adequate contrast between the raw BANG gel, the path of each 2 Gy beam and the intersection of the 2 beams. A second low dose RT planning CBCT scan was performed for targeting the calculated point of the intersecting beams.

#### Registration of the RT planning MR and CT images

As the CBCT image showed the glass of the vial as well as the BANG gel, the outer surface of the gel was used to guide the registration. From the CBCT image volume, only the gel, and not the glass vial, was segmented using the ITK-Snap (ITK-SNAP) region growing tool [24]. The MR image only showed the gel so the outer edge of the image was sufficient to drive the registration. The surfaces of these two gel masks were converted into a set of points which were aligned using a finite iterative closest point algorithm in MATLAB (The MathWorks, Inc., Natick, Massachusetts, United States). The transformation between the two sets of points was used to register the MR image volume to the CBCT image volume. The BANG gel samples were rotationally asymmetric volumes so that fiducial markers were not necessary for the MR-to-CBCT co-registration. The CT image defined the position of the object within the RT system and the combined CBCT-MR image then defined position of the intersecting beam target within the RT system.

#### RT planning on the intersecting beam target

The operator identified the centre of the intersecting beams in 3 dimensions using the Muriplan software. This isocentre was used to define the centre of rotation for a conical arc treatment, delivered at a 45 degree oblique angle, using a 5 mm round collimator and a 360˚ rotation of the beam with respect to the sample. A dose of 4 Gy was delivered to the rotational isocentre.

#### Assessment of RT planning accuracy by MRI

The same CE-FAST MRI scan was acquired after both the delivery of the target formation and the target treatment scans. The images were segmented using ITK-Snap [24] and any positional offsets between the centres of the intersecting beams and the treatment spheres were determined.

### In vivo stability of body position

Whilst BANG gel tests offered a means for establishing the degree of mechanical accuracy of RT treatment delivery, they did not present the problems associated with delivery of RT to a living animal. Assessments were made of the stability of animal and organ positions within the body as a function of time (intrinsic motion) and that of animals being moved repeatedly between MRI and IGRT treatment systems (extrinsic motion). The intrinsic motions, including respiration, peristalsis, bladder filling, and body sag, are unavoidable and present the fundamental limit to the accuracy with which RT can be delivered. Extrinsic body motions may be introduced by a combination of imperfect cradle design and incompetent transfer of the animal in its cradle from the MR scanner to the RT system.

#### MRI methods

Scans were performed at 7.0 T with a respiratory-gated 3D balanced SSFP (bSSFP) sequence [25] with TR 3.268 ms, TE 1.134 ms, FOV 64×32×32 mm^3^, matrix 256×128×128 and a 15° degree flip with a 16 μs hard pulse [26, 27]. Scans were performed with and without RF phase alternation in order to generate a maximum-intensity projection image with reduced banding artefacts from local magnetic field inhomogeneities. Scan time was respiration-rate dependent but lasted typically about 3 minutes.

#### Image registration

All rigid body registrations between sequentially acquired MR images were performed using the ‘imregister’ function built into MATLAB.

#### Evaluation of intrinsic motions

The bSSFP scan was repeated every 5 minutes for approximately 30 minutes for 5 CBA mice and to monitor stability of body position in the absence of external influences. Motion was assessed by taking the difference image of sequentially acquired images in ImageJ (author Wayne Rasband, Research Services Branch, National Institute of Mental Health, Bethesda, Maryland, USA) [28].

#### Evaluation of extrinsic motions

The bSSFP scan was performed prior to the animal in its cradle, being moved to the IGRT system, where a dummy CBCT-IGRT procedure without x-ray delivery, was performed. The animal was then returned to the MRI system and this cycle was repeated 4 further times. Using the cradle hardware described, it was not possible to return the animal and cradle to identical positions within the magnet with each consecutive loading, and rigid body registrations were performed to assess this. Deformable registration methods could not be used here as they would have concealed the residual motions. Motion was assessed by taking the difference image of sequentially acquired images in ImageJ [28].

### In vivo MR-IGRT of the adrenal glands and histological validation by γH2AX staining

#### MRI methods

The bSSFP scan as described in the section ‘In vivo stability of body position’ was used.

#### RT planning CBCT image

An RT planning CBCT image was acquired as described in the ‘Mechanical accuracy testing using BANG polymer gel dosimeters’ section but 50 kVp and not 70 kVp X-rays were used which resulted in a typical dose per CBCT scan of 22.7 ± 1.1 mGy.

#### Image registration

First a rigid body registrations between MR to CBCT images was performed using the ‘imregister’ function built into MATLAB. A further non-rigid registration step was required to compensate for the non-linear spatial distortions inherent in MR imaging, and was performed using the ‘MIND’ algorithm [14]. The bSSFP images had sufficient resolution and contrast to identify the adrenal glands and to drive the MR to CT image registration reliably.

#### MR-IGRT methods

In 4 CBA mice, positioned supine in the cradle, the bSSFP scan was used to identify the adrenal glands. This MR image was co-registered with the RT planning CT as described above and the combined CT-MR image was used to guide the RT treatment delivery. In 2 mice the left adrenal gland was targeted while the right adrenal was targeted in the remaining 2 mice. MRI-guided radiotherapy (10 Gy dose) was achieved using a 2 or 3 mm circular field (the collimator size was matched to the imaged size of the adrenal gland), with the beam delivered as a single beam passing anterior-to-posterior through the body.

#### Tissue preparation for γH2AX staining and confocal microscopy

Mice were not allowed to recover from anaesthesia and were culled 30 minutes post RT. Both adrenal glands were removed from each body, and histology, microscopy and analysis was performed by an operator blinded to the treatment schedule. The adrenal glands were collected, fixed in 10% neutral buffered formalin for 24 h, transferred to 70% ethanol for 24 h, and embedded in paraffin. The entire adrenals were cut into 5 micrometre sections and the sections were allowed to dry overnight. Following antigen retrieval (20 min, 100 ˚C, 10 mM sodium citrate buffer, pH6), the sections were permeabilized (1% triton X-100 in phosphate-buffered saline (PBS), 10 min, room temperature), blocked (Mouse-on-Mouse block (Vector Laboratories, UK), 5 drops/2.5 ml, overnight, 4 ˚C and 2% bovine serum albumin in PBS, 2 h, room temperature), and incubated with monoclonal anti-γH2AX antibodies raised in mouse (overnight, 4 ˚C, 1:500; p-yH2AXSer139 in the formulation of clone JBW301, Millipore, UK). An Alexa-594 labelled horse anti-mouse monoclonal antibody was used as secondary antibody (1h, room temperature, 1:500; DI-2488, Lot ZB0318, Vector Laboratories, UK), and DAPI (1:1000, BD Pharmingen, UK) was used for nuclear staining. Sections were mounted with Vectashield hardset antifade mounting medium (Vector Laboratories, UK), and images acquired using confocal microscopy (Leica TCS). Haematoxylin and Eosin (H&E) staining was performed as described before [29] and slides were scanned using Aperio Slide Scanner (Leica Biosystems, UK). All chemicals, unless stated otherwise, were purchased from Sigma-Aldrich UK.

### In vivo MR-IGRT of neuroblastoma xenografts

#### MRI methods

MRI imaging, using the bSSFP scan, was performed as described in the section ‘In vivo stability of body position’.

#### RT planning CBCT image and Image registration

CBCT imaging and subsequent image registration was performed as described in the section ‘In vivo MR-IGRT of the adrenal glands and histological validation by γH2AX staining’.

#### MR-IGRT methods

NOD scid gamma mice (NOD.Cg-*Prkdc*^*scid*^ *Il2rg*^*tm1Wjl/*SzJ^, Charles River, UK) were used as a host to generate human neuroblastoma tumours after subcutaneous injection of 5x10^6^ SK-N-SH cells (ATCC-HTB-11; LGC Standards, Middlesex, UK) in Matrigel (1:1 culture medium/Matrigel; BD Biosciences, Oxford, UK) in the right flank [30]. The tumour was excised 5 weeks post inoculation and following homogenisation, a crude suspension of SK-N-SH human neuroblastoma xenograft cells was injected into the right flank of BALB/c nu/nu mice (Charles River, UK; n = 8). Tumours entered the study when they reached 8 mm GMD (gross maximum diameter), approximately 7 weeks post inoculation. To monitor tumour response to radiotherapy, 5 mice underwent MR-IGRT and 3 were used as a control.

The bSSFP MRI scan was used to monitor tumour growth for entry into the study, for enabling MR-IGRT, and for monitoring the response to treatment. The MR image was co-registered with the RT planning CBCT as described above and the combined CBCT-MR image was used to guide the RT treatment delivery. A single 5 Gy beam was applied using the most appropriate collimator depending on the size and shape of the tumour to ensure complete coverage. In some mice, due to the limited number of collimator sizes available, more than one field was used in order to cover the whole tumour whilst ensuring minimal exposure of surrounding normal tissues. Moreover, minimal overlap of the two RT fields could be applied as a result of the sharp beam edges of the SARRP IGRT system [6, 12]. In order to maximise efficiency and throughput two cradles were used: while one mouse was being irradiated, the next mouse was undergoing MRI prior to RT. Mice were culled when tumours reached 800 mm^3^ as measured by region-of-interest analysis of the MR image, performed with ImageJ.

### In vivo MR-IGRT of spontaneous pancreatic tumours in KPC mice

#### MRI methods

MRI imaging, using the bSSFP scan, was performed as described in the section ‘In vivo stability of body position’. Additionally, a constant TR respiratory-gated 2D multi echo CPMG sequence using SPLICER [31] with TR 4.38 s, 8 echoes with first TE and echo spacing 6.856 ms, FOV 64×32 mm^2^, matrix 192×96, 72 contiguous 0.33 mm thick slices, was used to aid tumour identification in KPC mice. Scan time was respiration-rate dependent but lasted typically about 10 minutes.

#### RT planning CBCT image

CBCT imaging was performed as described in the section ‘In vivo MR-IGRT of the adrenal glands and histological validation by γH2AX staining’

#### Image registration

Image registration was performed as described in the section ‘In vivo MR-IGRT of the adrenal glands and histological validation by γH2AX staining’. Of the two MR imaging sequences used, only the bSSFP was found to have sufficient resolution for reliable MR to CT registration. However, the transformation between bSSFP and CPMG is fixed (since they are both acquired on the same scanner during the same imaging session), and so the transformation found between bSSFP and CT was applied to the CPMG images. The registered MR images were interpolated to the same resolution as the CT images and stored such that the RT planning software imported them as if they were the CT images.

#### MR-IGRT methods

The KPC model was used because the PDAC tumour is hard to localise without imaging and because it lies in the immediate vicinity of a number of highly radiosensitive organs including stomach, spleen and intestine. KPC mice develop invasive disease at age 47–355 days and survive with further development of pancreatic ductal adenocarcinoma over the next 2 and 10 months [16]. KPC mice were screened for pancreatic tumours every 3 weeks using bSSFP and CPMG MRI sequences. When tumours were identified at a diameter of ca. 4–5 mm mice were entered into the study (n = 5). The CPMG scan consistently gave the best tumour-to-normal tissue contrast and guided the localisation of the tumour centre. A dose of 2 Gy was delivered using a coronal arc beam centred on the tumour with the beam 45° to the vertical and the mouse rotated through 360°; the collimator and associated field size was chosen to ensure full coverage of the tumour while sparing surrounding tissues. Again, 2 cradles were used to improve time efficiency of the MRI-guided radiotherapy procedure.

#### Dose-volume histograms for different RT plans

In addition, the tumour and surrounding OARs were contoured slice-by-slice on the co-registered CBCT-MRI images. The OAR included stomach, bowel (small and large bowel; contoured from underneath the diaphragm up to 3 mm below the inferior PTV margin), liver, spleen, right and left kidney. The RT dose distributions and associated dose-volume histograms (DVH) for each organ were calculated for four different RT plans. The plans were as follows:

1. 45° Arc: RT planning using a single arc administered with the gantry fixed at 45 degrees and the mouse rotating through 360 degrees;
2. 0° beam: RT planning using a single static beam from 0°;
3. 30°+60° arc: RT planning using two conical arcs, one with the gantry at 30 and one at 60 degrees, both rotating through 360 degrees;
4. 2x120° arcs: RT planning using two arcs keeping the mouse static, and rotating the gantry from 0 to 120 degrees one on each side (equivalent to moving the gantry from -120 to +120 degrees).

The standard dose-volume histograms were extracted using MATLAB and plotted using the GraphPad PRISM (Version 6, GraphPad Inc., US).

## Results

### Mechanical accuracy testing using BANG polymer gel dosimeters

**Fig 2:**
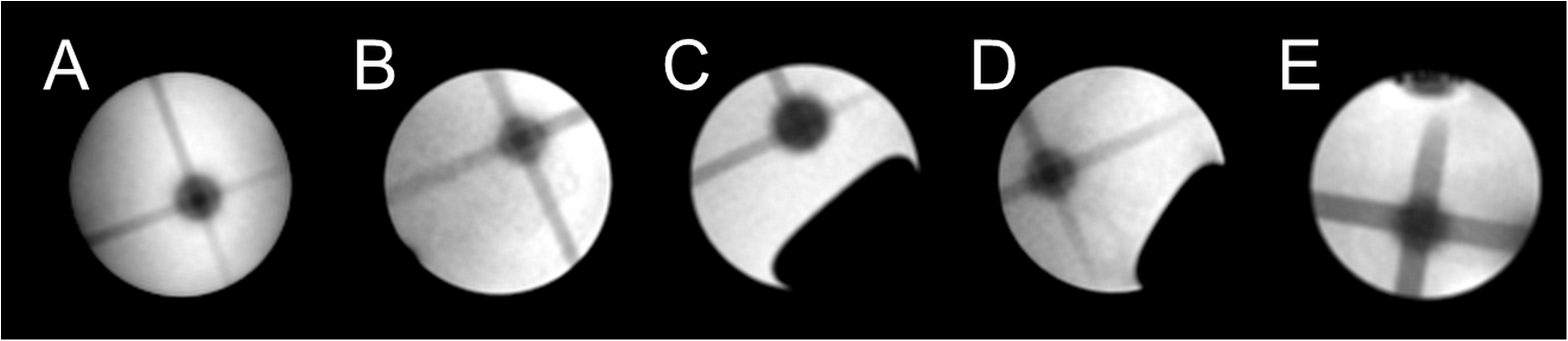
MR-IGRT of a painted feature in a BANG gel. (A)—(E): Transversal view of MR-IGRT for the 5 tested BANG gels. A target volume was created by a pair of orthogonal 2 mm diameter (A, B, C, D) or 4 mm diameter (E) circular beams (volume 4.2 or 33.5 mm^3^) and targeted following MR-IGRT using a conical arc, delivered at a 45 degree oblique angle and using a 5 mm round collimator.

MR-IGRT targeting efficiency was evaluated by studying the 3D dose distribution in BANG gels. The MRI-IGRT targeted 4.2 mm^3^ contrast volume could not be distinguished in CBCT but was readily visualised by MRI and enabled the delivery of MR-IGRT to the centre of the MR-visible contrast volume, as shown in Fig 2. The centre of the conical arc aligned with the centre of the preformed sphere target with a mean offset of 0.56 ± 0.18 mm (or 2.0 ± 0.7 pixels; n = 5).

### In vivo stability of body position

#### Evaluation of intrinsic motions

**Fig 3:**
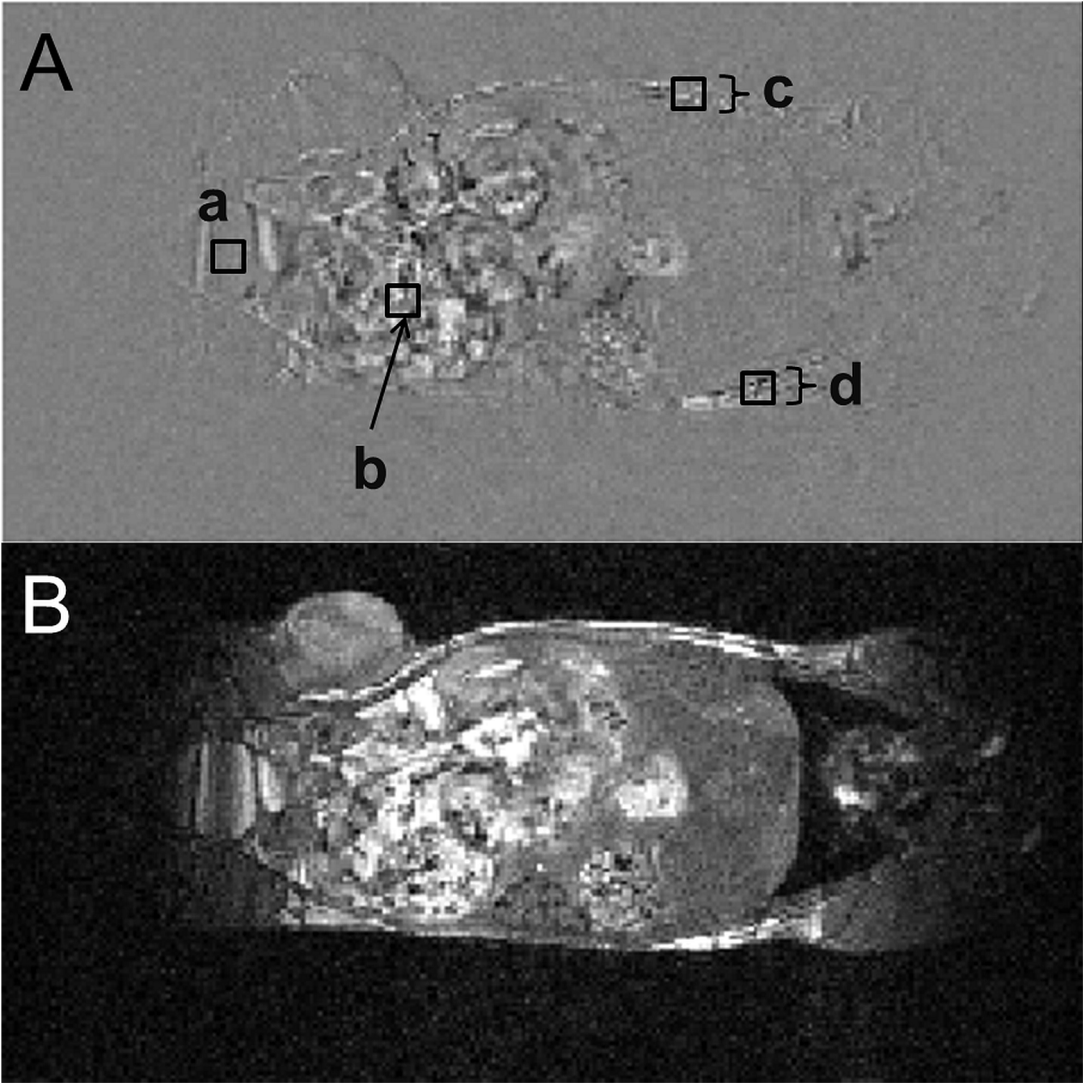
In vivo stability of body position: evaluation of intrinsic motions. (A) Intrinsic motion as shown by taking the difference of the first and last image of a 30 minute acquisition. Bladder filling (a), gastrointestinal motility (b) and a general gravity induced body droop of up to 4 pixels (1 mm) (c, d) can be observed. The differences in signal intensity, expressed as the percentage of the first image, are 8%, 51%, 3% and 3% for regions a, b, c and d, respectively. (B) The corresponding anatomical image is shown.

A representative difference image for the intrinsic motion is presented in Fig 3A. Significant gastrointestinal motility, bladder filling and a general gravity induced body droop of up to 4 pixels were observed during the 30 min time frame.

#### Evaluation of extrinsic motions

**Fig 4:**
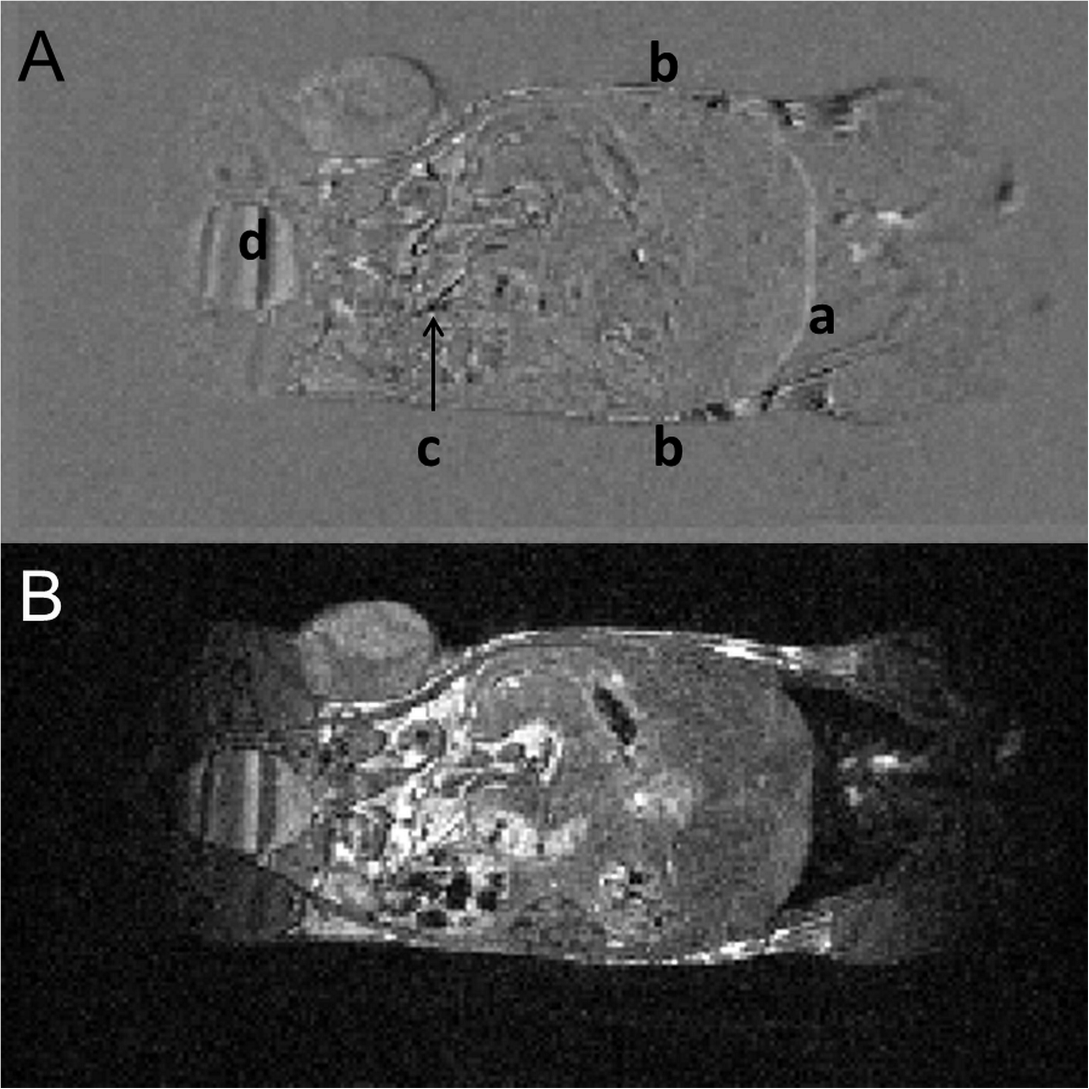
In vivo stability of body position: evaluation of extrinsic motions. (A) This represents the worst case image for extrinsic motion as shown by taking the difference image of the MR images produced before and after 4 cycles of transfer of the mouse in the cradle between the MR- and IGRT systems. Low level distortions can be observed at the diaphragm (a) and at the surface (b) of the mouse. Gastrointestinal motility (c) and bladder filling can also be observed (d). (B) The corresponding anatomical image is shown.

The difference image of MR scans performed before and after 5 cycles of transfer of the mouse in the cradle between the MRI scanner and the IGRT system is presented in Fig 4, illustrating the sum of intrinsic and extrinsic motions. It must be noted that only rigid transformation was applied to correct for positional errors between subsequent MR acquisitions. Low level distortions can be observed at the diaphragm and at the surface of the mouse. However, these mismatches are within 2 pixels (equivalent to 0.56 mm) for the diaphragm and 1 pixel (i.e. 0.28 mm) for the outer surface of the mouse and are within the MR-IGRT targeting error as described for the BANG polymer gel dosimeters.

### In vivo MR-IGRT of the adrenal glands and histological validation by γH2AX staining

Although the BANG gel tests established the degree of accuracy of RT treatment delivery, they do not represent the in vivo situation where accurate delivery of RT to the living body is crucial and where non-rigid body registrations are part of the workflow.

**Fig 5:**
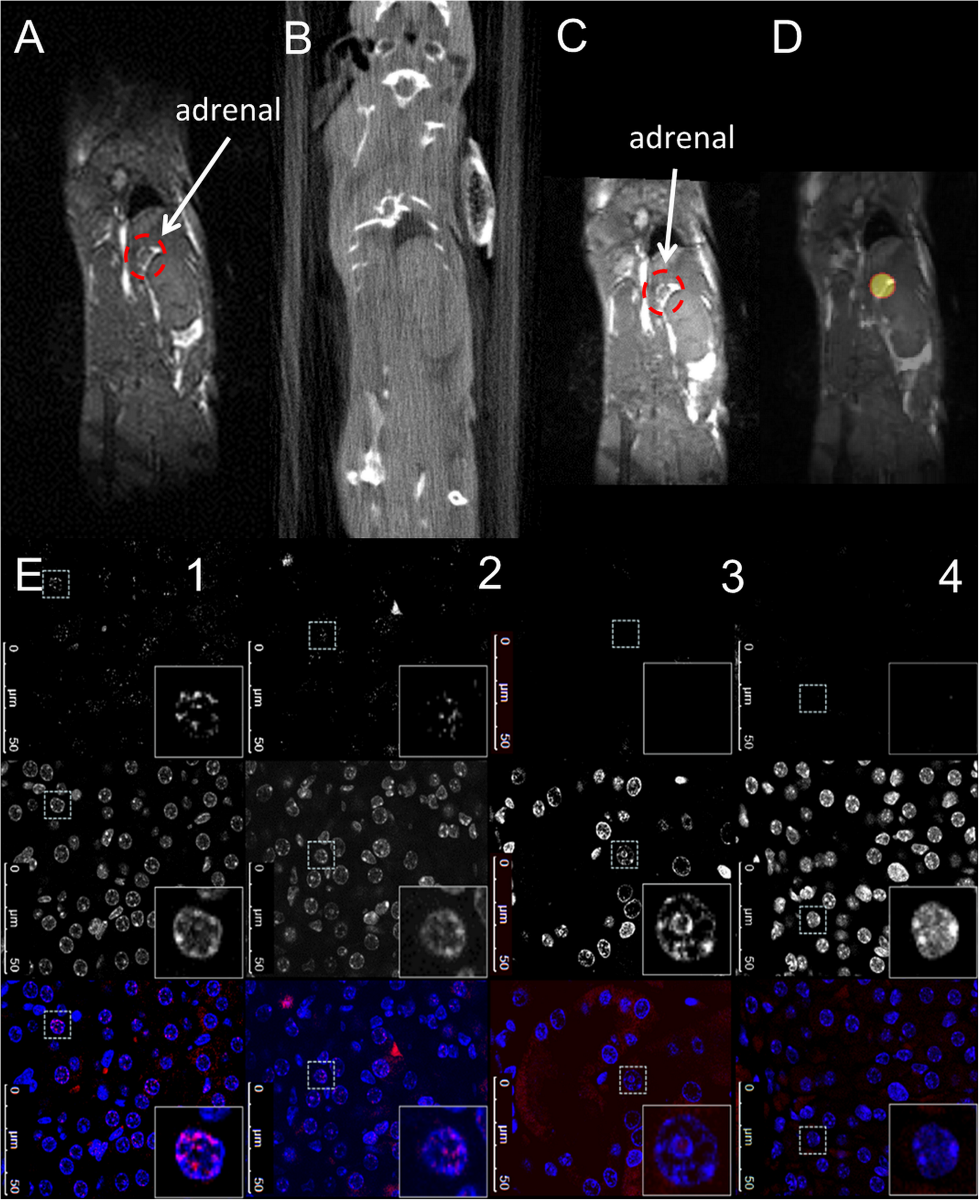
In vivo MR-IGRT of the adrenal glands and histological validation by p-γH2AXSer139 staining. (A) respiratory-gated bSSFP MR image; (B): RT planning CBCT image; (C) CBCT-MR image to guide the RT treatment delivery; (D) treatment plan for the adrenal; (E) The top row shows fluorescent images of p-γH2AXSer139 that were acquired for 2 regions within the irradiated adrenal (column 1 and 2), the proximal kidney area to the irradiated adrenal (column 3) and the non-irradiated contralateral adrenal (column 4). The sections were counterstained with DAPI to visualize nucleus (middle row) and the merged image is shown in the bottom row. Representative images are shown.

The contrast sensitivity of the CBCT was not sufficient to visualise the adrenals for IGRT planning. As a result, MR-IGRT was implemented. Respiratory-gated bSSFP MR allowed for fast imaging (86 ± 11 s/acquisition; approximately 3 minutes total) of the abdomen, and the adrenals could be easily localised. As a result, MR-IGRT of the adrenal gland was performed as shown in Fig 5A–5D. The MR-IGRT procedure took approximately 50 minutes for each mouse to complete.

MR imaging was successfully used to guide radiotherapy to one of the adrenals as confirmed by γH2AX staining, a marker of DNA double-strand breaks (Fig 5E). γH2AX foci analysis could distinguish the irradiated from the non-irradiated adrenal (Fig 5E, column 1 and 2 versus column 4). Moreover, the adjacent kidney to the irradiated adrenal showed background levels of γH2AX foci (Fig 5E, column 1 and 2 versus column 3).

### In vivo MR-IGRT of neuroblastoma xenografts

**Fig 6:**
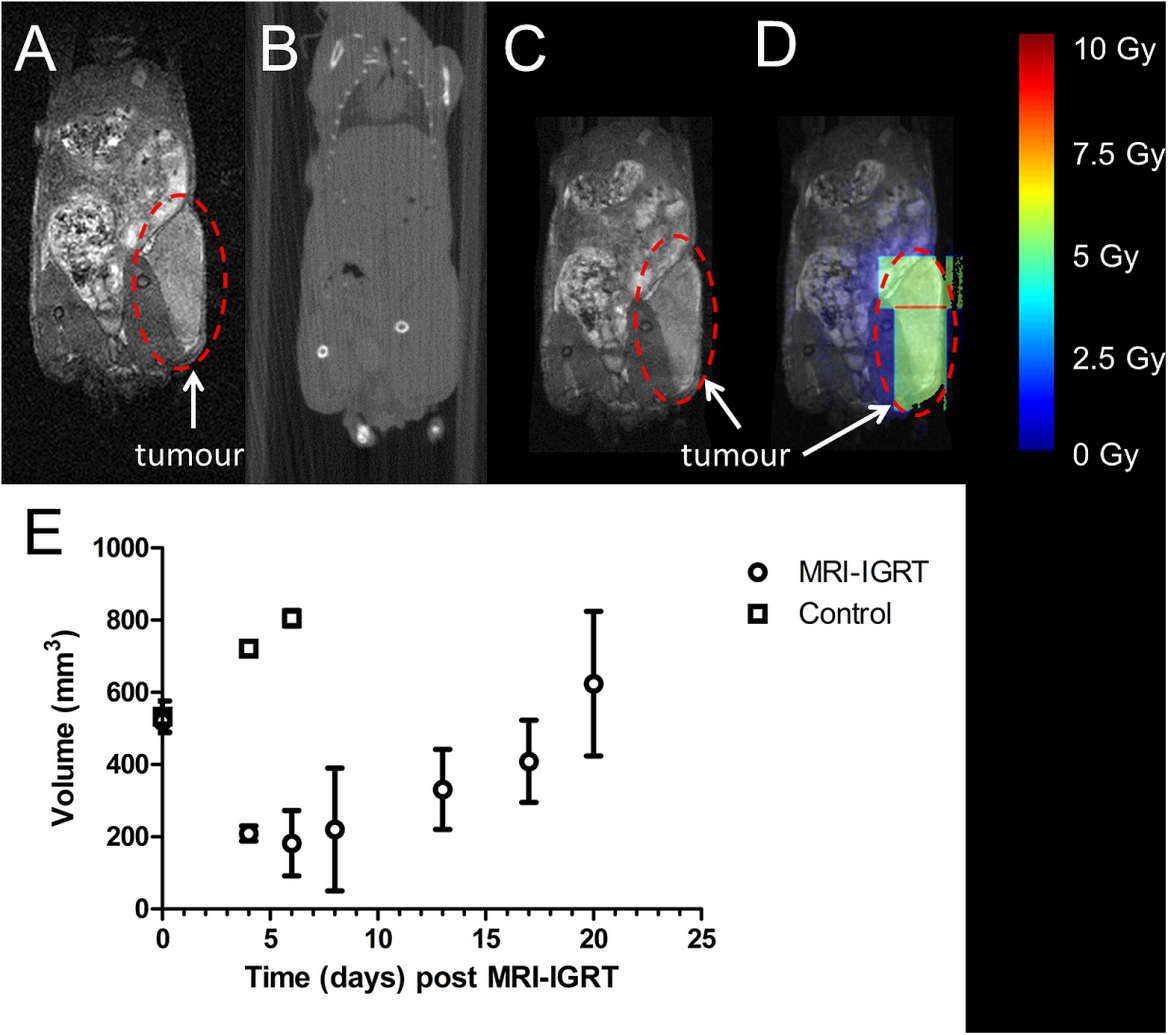
In vivo MR-IGRT of neuroblastoma xenografts. (A) respiratory-gated bSSFP MR image; (B): RT planning CBCT image; (C) CBCT-MR image to guide the RT treatment delivery; (D) dose distribution for the targeted treatment plan for a large tumour based on CBCT image to guide the RT treatment delivery: 2 radiotherapy fields with minimal overlap were used to cover the whole tumour whilst ensuring minimal exposure of surrounding normal tissues. The tumour is indicated within a dashed line; (E) Tumour volume of MRI-IGRT treated (5 Gy; n = 5) and non-irradiated control mice (n = 3). Values are expressed as mean ± standard deviation.

Fig 6 presents the MRI-guided radiotherapy plan to irradiate a SK-N-SH neuroblastoma xenograft. As the MR image (Fig 6A) shows, these tumours are readily apparent but tend to grow inwards rather than outwards and they are not discernible using the CBCT image (Fig 6B). The respiratory-gated bSSFP scan delineated these tumours clearly and within 10 minutes of induction of anaesthesia. Additionally, throughput was maximised by using 2 support cradles as outlined before. As a result, MRI-guided radiotherapy of 6 animals could be completed within 1h45min as the time to perform bSSFP fitted well within the time required to perform CBCT-IGRT.

As proof of principle, these neuroblastoma xenografts were treated by MR-IGRT and tumour volume was followed over time. Tumour regression was observed during the initial 5 days post radiotherapy. However, recurrence was observed on average at day 8 with the endpoint reached at day 20 (Fig 6E).

### In vivo MR-IGRT of spontaneous pancreatic tumours in KPC mice

**Fig 7:**
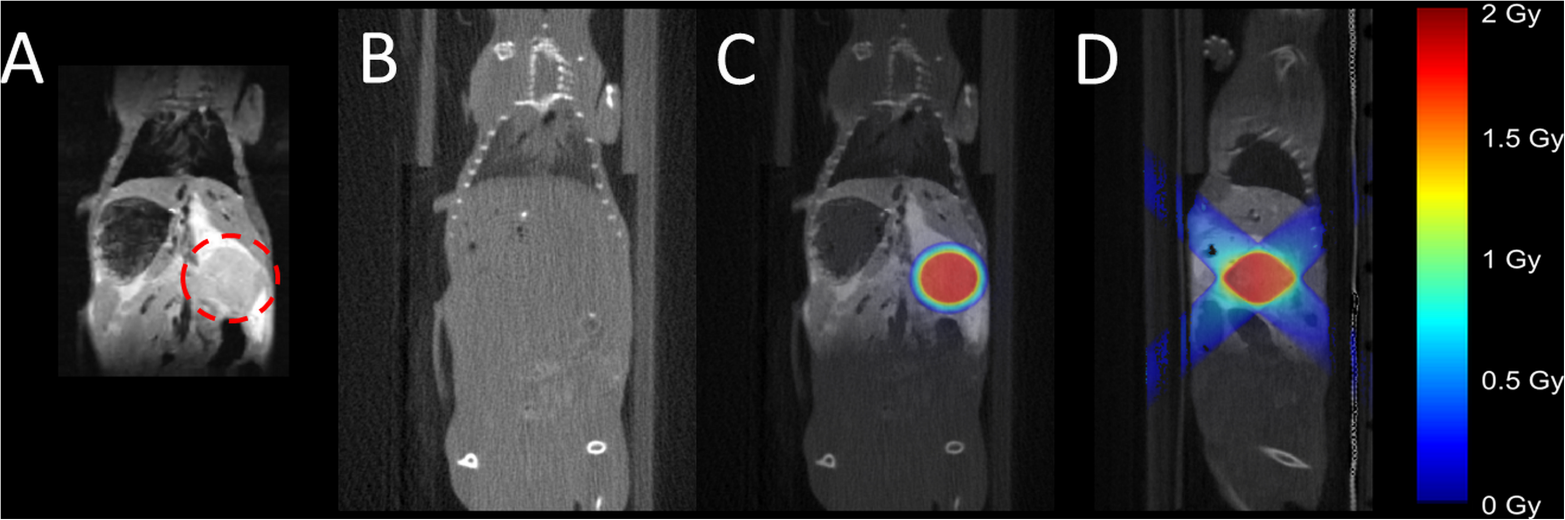
In vivo MR-IGRT of spontaneous pancreatic tumours in KPC mice. (A) respiratory-gated bSSFP MR image; (B) RT planning CBCT image; (C) coronal and (D) sagittal view showing dose distribution (prescribed 2 Gy) based on CBCT-MR image to guide the RT treatment delivery. The colour heat map indicates the RT doses.

**Fig 8:**
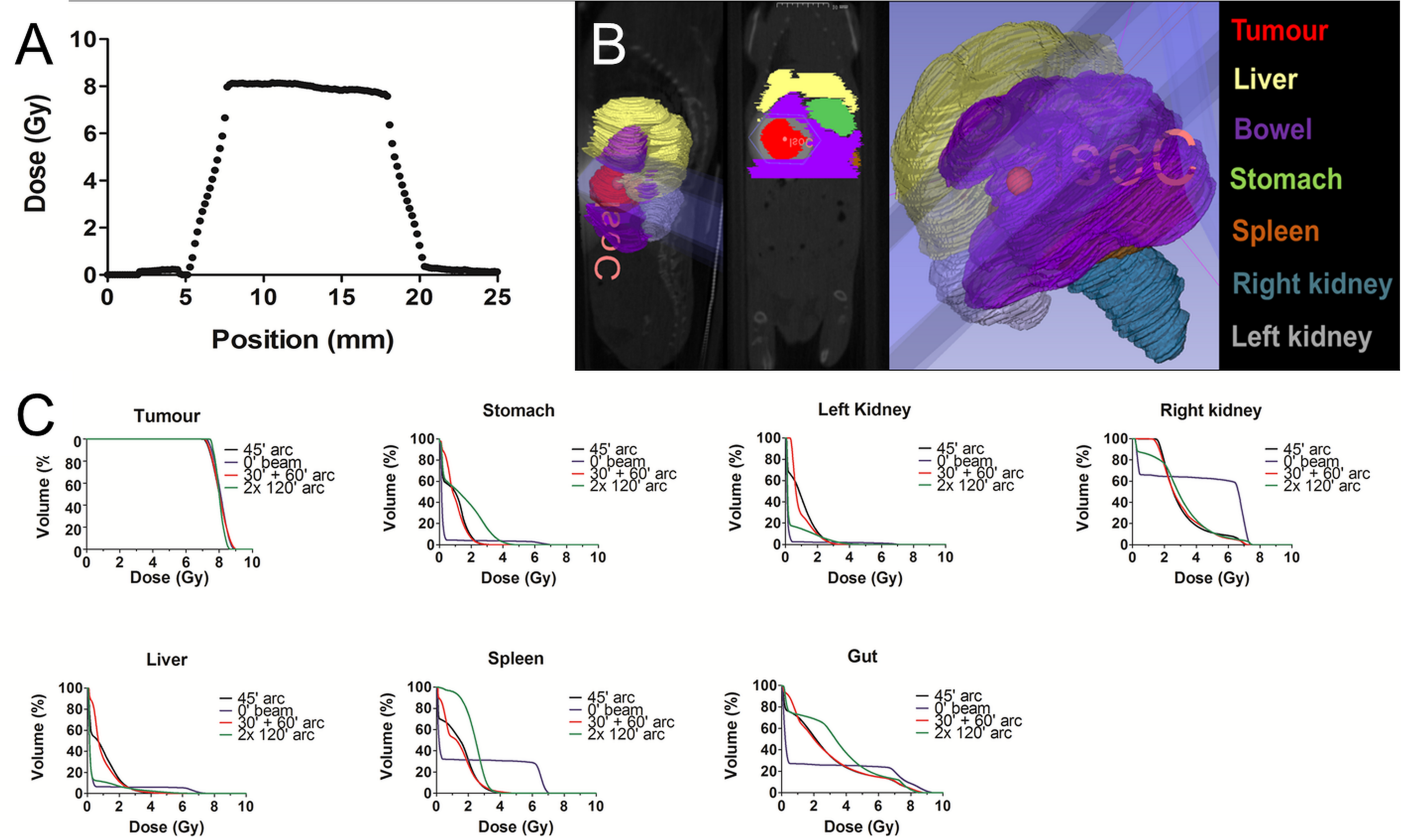
Determination of the optimal RT plan for intra-abdominal tumours in a mouse model of PDAC using a 10 mm diameter circular field. (A) Planned dose profile for a pancreatic tumour determined at the isocentre, perpendicular to the angle of rotation (0°) and perpendicular to the sagittal axis of the mouse. (B) Illustration of MR image-based delineated tumour and organs at risk on the sagittal (left panel), coronal (middle panel) and 3-dimensionally, as indicated. (C) Mean dose-volume histograms for a prescribed single dose of 8 Gy showing the dosimetric distribution of four radiotherapy plans (45° Arc, 0° beam, 30°+60° arc and 2x 120° gantry arcs, respectively) to the intra-abdominal pancreatic tumour and the organs at risk in the KPC mouse model, as indicated.

Localisation of spontaneous pancreatic tumours in the KPC model of PDAC can be achieved by combining respiratory-gated bSSFP and CPMG MR imaging and the RT dose plan was produced as shown in Fig 7. Again, the tumour was inseparable using CBCT (Fig 7B) but well resolved with MRI (Fig 7A) that facilitated target delineation and subsequent RT plan following a prescription of 2 Gy (Fig 7C and 7D). Fig 8A illustrates adequate dose coverage of the pancreatic tumour.

It currently remains unclear which is the optimal RT plan for irradiating intra-abdominal tumours in the KPC mouse model of PDAC. For that purpose, the tumour and OARs were contoured and the dose distribution to the tumour and OARs examined by plotting the dose-volume histograms for four different plans (45° Arc, 0° beam, 30°+60° arc and 2x120° arcs, respectively). A dose-volume histogram example for a pancreatic tumour located on the upper right side of the abdomen is shown in Fig 8C. All four plans achieved adequate and comparable dose coverage of the pancreatic tumour. As expected, a single vertical static beam (0° beam) resulted in worse dose distribution to the unilateral (right) kidney and spleen but slightly superior profile with better sparing of the contralateral (left) kidney compared to the other three plans. Interestingly, these three plans (especially 2x120° arcs) resulted in exposure of larger volume of stomach and bowel to the lower RT dose range (0 Gy up to 5 Gy) compared to 0° beam. No major differences were identified in the dose distribution to the liver among the four tested plans.

Finally, it important to note that throughput can be maximised by using 2 support cradles simultaneously with the current animal undergoing RT whilst the next animal to undergo RT is being scanned under MRI. As a result, MRI-guided radiotherapy of 6 animals bearing PDAC tumours could be completed within 2.5 h as the rate determining step is the duration of the MRI procedure (approximately 25 minutes), rather than the entire procedure (approximately 40 minutes). As both the bSSFP and CPMG acquisitions were needed to accurately identify the spontaneous PDAC tumours, throughput was slightly slower as compared to the xenografts which could be operated with a turnaround time of approximately 15 minutes per mouse.

## Discussion

We describe here a MRI-guided radiotherapy method that takes advantage of the strengths of each of its components: MRI for soft tissue contrast and target identification, CBCT for accurate dose calculation and IGRT for accurate, collimated X-ray beam delivery. Each step of the proposed MR-IGRT procedure was validated: from in vitro BANG gel tests to complex in vivo targeting of abdominal organs, such as spontaneous pancreatic tumours in the KPC transgenic mouse model.

A phantom study using BANG gel dosimeters was performed to validate the mechanical accuracy of the MR-IGRT procedure. Such gels are radiologically soft-tissue equivalent and are used routinely in the clinic to validate IGRT procedures [18]. Similarly, high resolution 3D dose distribution measurements were obtained and indicated that a target, invisible for CBCT, but clearly delineated using MRI, could be targeted with an error of approximately 2 pixels (0.56 mm). This error results from multiple sources, including distortions arising from the image formation processes, the digitisation of the target, errors consequent to the registration process as well as the operator bias inherent to the use of trained operators prescribing the centre of rotation manually. Although the centre of the conical arc treatment did not perfectly align with the centre of the target, the 0.6 mm targeting accuracy was comparable with a 3D offset of 1 mm reported in a similar study using BLI as an imaging tool [32]. Therefore, automated image-guided isocentre definition has the potential to further increase the mechanical accuracy of MR-IGRT.

However, phantom systems such as BANG gels do not reflect the in vivo situation for which gastrointestinal, respiratory, cardiac and other motions are present during image formation, RT planning and RT delivery. To make the MR-IGRT work in vivo, subject immobilisation throughout the whole procedure is crucial as accurate registration of MRI-to-CT images will depend on the magnitude of intra- and/or inter-scan displacements [10, 13]. To minimise the effects of these an MR- and CBCT-compatible animal support cradle was developed. This cradle features head fixation via a tooth bar assembly anaesthetic gas delivery, respiratory monitoring and homeothermic temperature control. With appropriate operator care, the animal’s position within the cradle remains almost constant throughout the procedure. The latter was formulated by Thorek et al. as a prerequisite for animal image registration [8, 13]. Instrinsic motions could be attributed solely to gastrointestinal contractions and general body droop whilst extrinsic motions, due to the actual transport of the cradle between systems, were limited to 0.5 mm, approximately the same as the measured error in RT planning accuracy derived from the validation tests using BANG gels. As a result, co-registration between MR and CBCT images becomes straightforward and meaningful.

The first in vivo MR-IGRT test included targeting one adrenal gland with a size-matched collimated beam whilst the other adrenal was spared. The adrenal glands are small organs (approximately 1.5–3 mm in diameter) that are located on both sides of the body in the retroperitoneum, cranially to the kidney. They cannot be identified using CBCT but are readily apparent in MR images. Any failure in any part of the MR-IGRT procedure would result in an absent or partial distribution of the DNA double-strand break marker γH2AX foci, in the MR-IGRT targeted adrenal glands. While the cell nuclei of the non-irradiated adrenal and the kidney adjacent to the irradiated adrenal showed very few γH2AX foci, the foci were abundant and homogenously distributed in the irradiated adrenal. This, together with the fact (1) that the X-ray beam was collimated with dimensions matched to those of the adrenal gland and (2) that the SARRP platform penumbra is sharp [6, 12], indicated that the MRI-guided procedure was also accurate in an in vivo setting.

Further validation was performed by assessing the response-to-treatment of a neuroblastoma xenograft model. These tumours are invisible on CT images and show an endophytic growth pattern, making them nearly undetectable when palpating the animal. These xenograft tumours are hard to irradiate with conventional large field, single radiation beam X-Ray cabinets, as the borders of the tumours are not discernible due to their non-solid structure. This increases the risk of either not irradiating the whole tumour or exposing normal tissue from surrounding organs to irradiation. Respiratory-gated bSSFP MRI could visualise these tumours easily and quickly after which RT could be delivered. As proof-of-principle for X-ray beam delivery, tumour growth was measured over time. As expected for the radiosensitive neuroblastoma, tumour regression was observed almost immediately which confirms the accuracy of the MRI-guided radiotherapy method [30, 33].

Preclinical radiobiological research in PDAC has been limited by mouse models that do not recapitulate the human biology and, more importantly, the significant technical challenges in establishing a platform that enables precise irradiation of such tumours in mice. The in vivo validation was concluded by using the KPC transgenic model of pancreatic cancer. As the pancreas is a diffuse organ in the mouse, these abdominal lesions are not readily identifiable by CT, and their exact location cannot be predicted. This not only makes it difficult to identify the lesions, but its abdominal location also poses challenges for imaging as any extra-cranial organ cannot be easily immobilised. Both respiratory-gated bSSFP and CPMG MRI acquisitions were used to identify the tumours in the abdomen. Moreover, the pancreatic tumours could be separated from the normal pancreatic tissues without the need for contrast agents; such agents can potentially influence the subsequent RT planning and delivery [6]. These advantages make our proposed MR-IGRT method more robust compared to, for instance, reversed-contrast imaging [8]. Once identified, the tumours could be easily targeted by the MRI-guided radiotherapy method. Importantly, high throughput can be easily achieved when two support cradles are used. If the MRI acquisitions fit within the 20 minutes needed for radiotherapy planning and execution, then a turn-around time of 30 minutes per animal can be achieved. This enables fractionated radiotherapy schedules for approximately 16 mice each day. Finally, MR image-based target contouring and treatment planning facilitated evaluation of dose distributions not only to the tumour but also to the OARs. We detected differences in dose distribution among the four different plans. This can be of particular interest for RT tolerance investigations as small and large bowel toxicity constitutes a dose-limiting factor for RT of PDAC [34] and will be investigated in future studies.

Further improvements to the entire procedure can be expected with the additional use of functional or molecular imaging techniques that identify tumour with a higher sensitivity than may be possible with anatomical MRI. However, the methods described are generally useful; the cradles described can be used in multiple imaging systems from multiple vendors, and the image registrations used *in vivo* were designed specifically to handle images acquired using multiple different modalities. We anticipate the use of PET and/or SPECT imaging in conjunction with both MRI and RT in order to treat tumours according to their PET or SPECT tracer uptake whilst ensuring minimum dose delivery to surrounding normal tissues as described by MRI. Implementation of this multi-modal image-guided RT workflow is a straightforward extension of the suite of techniques described in this report and will be investigated in further studies.

## Conclusion

The proposed MR-IGRT method presents a general solution to enabling robust, accurate and efficient targeting of extracranial organs in the mouse and can operate with a sufficiently high throughput to allow fractionated treatments to be given. Our preclinical MR-IGRT platform is expected to provide important insight on the radiobiology research of clinically relevant mouse models in the immediate future.

## Supporting information

S1 Fig A flowchart representing the overall workflow and design of the reported study. (TIF)
