# Supplementary figures and images for: An efficient and robust MRI-guided radiotherapy planning approach for targeting abdominal organs and tumours in the mouse
Source: PLoS One. 2017 Apr 28;12(4):e0176693. doi: 10.1371/journal.pone.0176693 (PMC5409175; doi:10.1371/journal.pone.0176693)

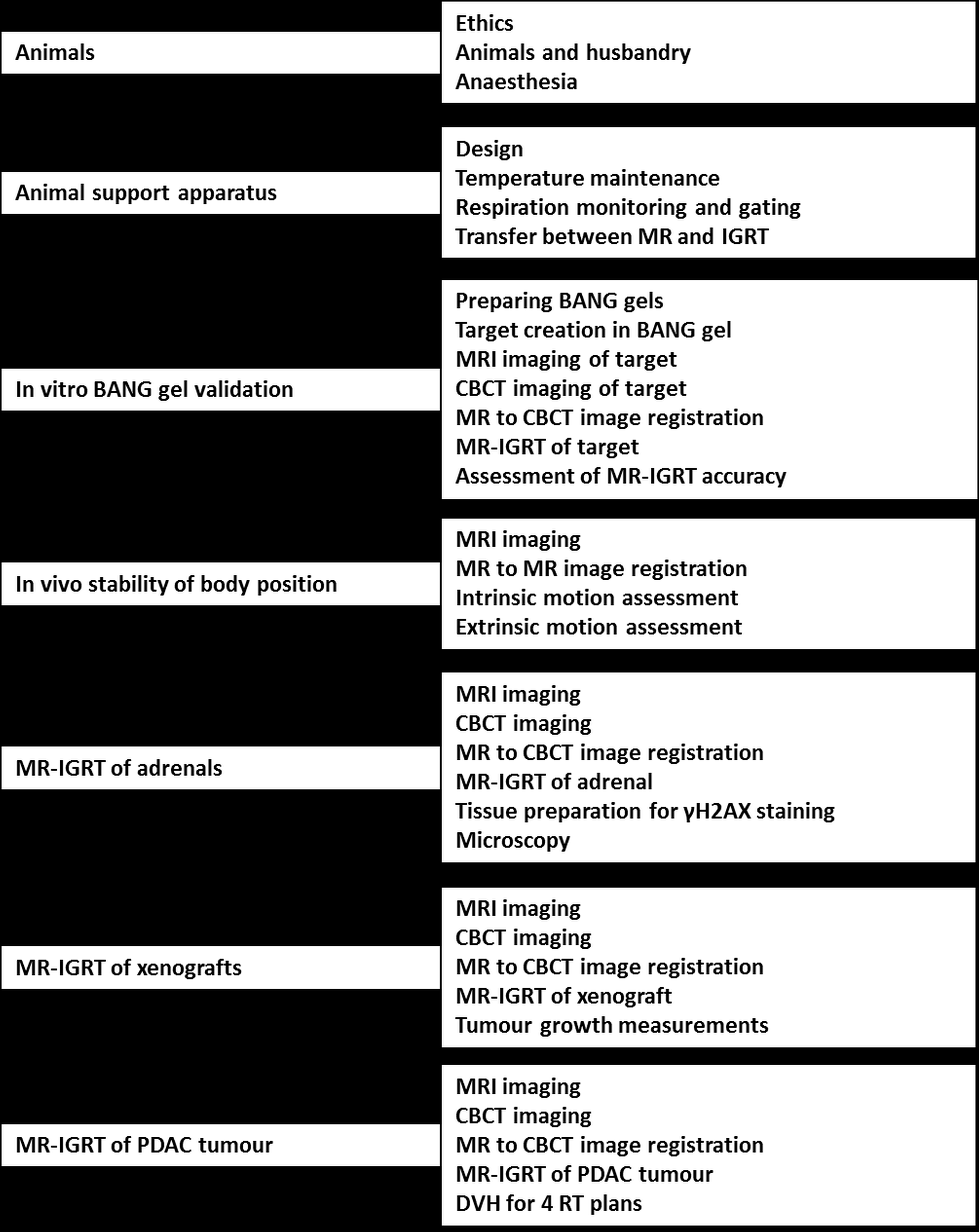

Supplement: S1 Fig — (TIF) [file pone.0176693.s001.tif]
